# A single-domain antibody-linked Fab bispecific antibody Her2-S-Fab has potent cytotoxicity against Her2-expressing tumor cells

**DOI:** 10.1186/s13568-016-0201-4

**Published:** 2016-04-26

**Authors:** Aifen Li, Jieyu Xing, Li Li, Changhua Zhou, Bin Dong, Ping He, Qing Li, Zhong Wang

**Affiliations:** School of Pharmaceutical Sciences, Sun Yat-Sen University, Guangzhou, China; Center for Cellular and Structural Biology, Sun Yat-Sen University, Guangzhou, China

**Keywords:** Bispecific antibody, Single domain antibody, Her2, CD16, Fab, Bacterial expression

## Abstract

Her2, which is frequently overexpressed in breast cancer, is one of the most studied tumor-associated antigens for cancer therapy. Anti-HER2 monoclonal antibody, trastuzumab, has achieved significant clinical benefits in metastatic breast cancer. In this study, we describe a novel bispecific antibody Her2-S-Fab targeting Her2 by linking a single domain anti-CD16 VHH to the trastuzumab Fab. The Her2-S-Fab antibody can be efficiently expressed and purified from *Escherichia coli*, and drive potent cancer cell killing in HER2-overexpressing cancer cells. In xenograft model, the Her2-S-Fab suppresses tumor growth in the presence of human immune cells. Our results suggest that the bispecific Her2-S-Fab may provide a valid alternative to Her2 positive cancer therapy.

## Introduction

The human epidermal growth factor receptors (HER) play important roles in cell growth and signaling. Mutations of these receptors can lead to tumorgenesis (Cooke 2000). Among the members of HER, HER2 (human epidermal growth factor receptor 2), which is also known as Her2/neu or ErbB2, has been extensively studied as activation of Her2 signaling pathways can lead to cell proliferation and tumorgenesis (Olayioye 2001; Yarden and Sliwkowski 2001; Citri and Yarden 2006). HER2 is amplified and overexpressed in 20~30 % of invasive breast carcinomas. Its overexpression can also be found in various human cancers, such as gastric, lung, ovary, bladder and kidney carcinomas (Slamon et al. 1987; Press et al. 1994; Daniele and Sapino 2009). Various therapeutic approaches including monoclonal antibodies (mAbs) have been developed to block Her2 activity to combat tumor growth (Keler et al. 1997; Ben-Kasus et al. 2009; Vasconcellos et al. 2013). Trastuzumab (Herceptin^®^), which binds to the HER2 ectodomain, is the first clinical approved antibody targeting Her2. Trastuzumab can directly inhibit Her2 activity and induce ADCC (antibody dependent cell mediated cytotoxicity) through its Fc fragment. It can induce clinical responses in Her2-overexpressing breast cancers, and prolong the patient survival when combined with the chemotherapy (Ranson and Sliwkowski 2002; Hudis 2007; Spector and Blackwell 2009). However, for many metastatic breast cancer patients with HER2 gene amplification, the tumors do not respond to or eventually escape the effect of the trastuzumab due to de novo and acquired resistance (Valabrega et al. 2007; Junttila et al. 2010).

To combat Her2 positive tumors and antibody resistance, one of approaches that have been tested is bispecific antibody (Shalaby et al. 1995; Turini et al. 2014). Bispecific antibodies can engage the immune cells directly to the tumor cells by having two different antigen binding sites, with one recognizing the tumor cells and the other recognizing the immune cells, usually T cells or NK (natural killer) cells. Bispecific antibodies can redirect the cytotoxic potential of T cells or NK cells to eliminate the tumor cells (Fischer and Leger 2007). Various bispecific antibody formats have been developed and investigated, including IgG based formats and single chain based formats, such as diabodies, minibodies and tandem single-chain variable fragments (scFvs) (Muller and Kontermann 2007; Chames and Baty 2009). As ScFv based antibodies have the potential issues with poor stability; and tendency of aggregation (Fischer and Leger 2007), we applied single-domain antibody in the construction of bispecific antibodies (Li et al. 2015). Single-domain antibodies (sdAbs) are derived from heavy-chain only antibodies naturally devoid of light chains (Hamers-Casterman et al. 1993). Single-domain antibodies (sdAbs) are small, stable, and well expressed in *E. coli* (Dumoulin et al. 2002), representing as ideal molecular building units for bispecific antibody construction (Turini et al. 2014).

Following our previous work (Li et al. 2015), the Her2-S-Fab (Single domain antibody-linked Fab) was constructed by linking a single domain antibody anti-CD16 VHH (Behar et al. 2008) to the C-terminal end of a Trastuzumab Fab. The Her2-S-Fab can be expressed and purified from bacteria. In *vitro* cell-based assays, the Her2-S-Fab can specifically kill cancer cells with over-expression of Her2 by engaging the NK cells. Comparing to Trastuzumab, enhanced tumor cell killing was observed. *In vivo* studies further showed that the Her2-S-Fab could suppress cancer progression.

## Materials and methods

### Fab design and protein purification

**Fig. 1:**
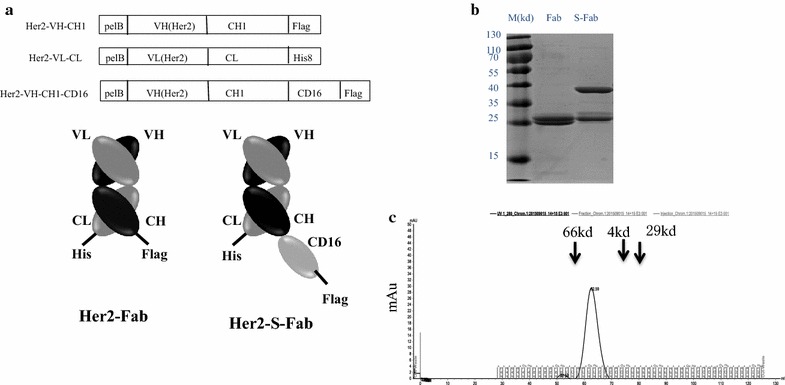
Her2-S-Fab purification from *E. coli*. **a** Bacterial expression constructs of Her2-S-Fab and control Her2-Fab. Each construct contains a pelB signal sequence for periplasmic expression; Co-expression the constructs of anti-Her2-VL-CL and anti-Her2-VH-CH1 leads to Her2-Fab; Co-expression the constructs of anti-Her2-VL-CL and anti-Her2-VH-CH1- anti-CD16 VHH leads to Her2-S-Fab; To facilitate protein detection and purification, a Flag tag or a his8 tag were added to the C-terminal of each construct. **b** The commassie blue staining results of Her-S-Fab after two step purification. *M*, molecular weight ladder, unit: kD; (**c**) gel filtration chromatography showed the size of Her2-S-Fab is approximately 65 kD

The constructs of Her2-S-Fab and control Her2-Fab are shown in Fig. 1a. By standard DNA cloning techniques, the Trastuzumab anti-Her2 VL-CL and VH-CH1 were first chemically synthesized based on previous reports (Sommaruga et al. 2011; Akbari et al. 2014) and cloned into the pET21a vector and pET26b vector. The VHH-CD16 (Behar et al. 2008) was then cloned into the c-terminal of Trastuzumab anti-Her2 VH-CH1 with similar strategy to previous reported (Li et al. 2015). A signal sequence pelB was added to the N-terminus for periplasmic expression (Spiess et al. 2013). The Her2-S-Fab was formed via the heterodimerization of VL-CL/VH-CH1-VHH (CD16), and the control Her2-Fab was formed via the heterodimerization of VL-CL/VH-CH1.

The periplasmic protein purification was performed as described before (Kwong and Rader 2009). Briefly, two plasmids encoding individual polypeptides were co-transformed into the BL21 (DE3) cells with proper antibiotics. Protein expression was induced and periplasmic extraction was performed. Control anti-HER2 Fab or Her2-S-Fab proteins were then purified from the combined sucrose and periplasmic fractions by Ni–NTA Agarose affinity (MCLAB, NINTA-300) and then anti-IgG CH1 affinity purification. Gel filtration was performed using GE Hiload 16/600 Superdex 200 pg as described by the manufacturer (GE). Gel filtration protein markers were from Sigma (MWGF1000).

### Cell lines and animals

HER2-positive cancer cell lines SKBR3 (human breast cancer cell), BT474 (human breast cancer cell), SKOV3 (human ovary cancer cell), MCF7 (human breast cancer cell), the HER2-negative cancer cell line MDAMB435, MDAMB468, the Chinese hamster ovary (CHO) were purchased from the Type Culture Collection of the Chinese Academy of Sciences, Shanghai, China. SKBR3, SKOV3 and MDAMB435 were cultured in Dulbecco’s Modified Eagle Medium (DMEM, Gibco, Life Technologies, China) with 10 % HI fetal bovine serum (Gibco, Life Technologies, USA) and 1 % Penicillin/Streptomycin (HyClone); BT474 and CHO were cultured in RPMI-1640 medium (Gibco, Life Technologies, China) with 10 % HI fetal bovine serum (Gibco, Life Technologies, USA) and 1 % Penicillin/Streptomycin (HyClone), MDAMB468 were cultured in L15 (Gibco, Life Technologies, China) at 37 °C in a 5 % CO_2_ humidified incubator.

Non-obese diabetic-severe combined immunodeficiency disease (NOD/SCID) mice were purchased and then housed in the animal experiment center of Sun Yat-sen University under sterile and standardized environmental conditions (20–26 °C room temperature, 40–70 % relative humidity, and 12 h light–dark rhythm).

### Blood cell fractionation

Peripheral blood mononuclear cells (PBMC) were separated from healthy volunteers’ blood using Ficoll density centrifugation (So et al. 2013). NK cells were extracted by EasySep™ human NK cell enrichment Kit (STEMCELL Technologies, Inc., Vancouver, Canada). The isolated NK cells were cultured in complete RPMI 1640 with 10 FBS and 1 % penicillin/streptomycin at 37 °C in a 5 % CO_2_ humidified incubator before the cytotoxicity assay.

### Flow cytometry analysis

When tissue culture cell confluence reaches 80–90 %, cells were digested with 0.25 % trypsin and collected. 1 × 10^5^ cells per sample were collected by centrifugation at 1500 rpm for 5 min and then washed with 3 ml of ice cold PBS + 0.1 % BSA twice. The pellet was re-suspended in 500 µl of ice-cold PBS + 0.1 % BSA. In each tube, add Transtuzumab (gift from Alphamab, Suzou), Her2-Fab, or Her2-S-Fab as primary antibodies respectively. Goat-anti-human IgG (H + L) Alexa Fluor 488 (Invitrogen, cat#A11013) was used as secondary antibody. Anti-HER2/neu-PE(BD, cat#340552) was used as control. Flow cytometry analysis was performed after washing the cells twice.

### Cytotoxic assays

Cytotoxicity assays were performed as described before (Li et al. 2015). Briefly, SKBR3, BT474, MCF7, SKOV3, MDAMB435, MDAMB468, CHO cells were used as target (T) cells. Human PBMCs or isolated NK cells without prior stimulation were used as effector (E) cells. 100 µl of target cells (5000 cells) was plate to each well in 96-well plates in triplicate. After a 12-h incubation, equal volumes of NK cells were added to each well at an E:T ratio of 10:1. The indicated concentrations of antibodies, which varied from 0.01 ng/ml to 10 μg/ml, were then added. After a 72-h incubation, cell viability was quantified using CCK8 reagent (Dojindo, CK04) according to the protocol of manufacturer. The survival rate (%) of target cells was calculated via the following formula: $\left[ {{{\left( {{\text{live}}\,{\text{target}}\,{\text{cells}}\left( {{\text{sample}}} \right){\mathbf{ - }}{\text{medium}}} \right)} \mathord{\left/ {\vphantom {{\left( {{\text{live}}\,{\text{target}}\,{\text{cells}}\left( {{\text{sample}}} \right){\mathbf{ - }}{\text{medium}}} \right)} {\left( {{\text{live}}\,{\text{target}}\,{\text{cells}}\left( {{\text{control}}} \right)} \right){\mathbf{ - }}{\text{medium}}}}} \right. \kern-\nulldelimiterspace} {\left( {{\text{live}}\,{\text{target}}\,{\text{cells}}\left( {{\text{control}}} \right)} \right){\mathbf{ - }}{\text{medium}}}}} \right] \times 100.$

### In vivo tumor growth inhibition assay

In vivo tumor growth assays were performed as described previously with modifications (Junttila et al. 2014).

Briefly, SKOV3 cells were harvested from cell culture, washed once with PBS, re-suspended in PBS, and mixed with PBMCs freshly isolated from healthy donors. Cell suspensions were injected subcutaneously into the right flank of NOD/SCID mice in a total volume of 0.2 mL/mouse with mixtures of 2 × 10^6^ SKOV3 cells and 1 × 10^7^ human PBMCs. Two hours after SKOV3/PBMC cell engraftment, antibodies or vehicle control (PBS) were administered intraperitoneally (*i.p.*). The animals were then treated daily over the following 6 days. The weight of mice was measured and the condition of mice was observed every day. Tumor volume was measured everyday with calipers in 2 perpendicular dimensions and was calculated using the formula (width^2^ × length)/2. All the results are presented as the arithmetic mean for each group.

## Results

### Purification of Her2-S-Fab and Her-Fab

Her2-S-Fab was designed by genetically linking an anti-CD16 VHH (Behar et al. 2008) at the C-terminal of anti-HER2 VH-CH1 (Fig. 1a). Anti-HER2VH-CH1-VHH and anti-HER2 VL-CL were cloned into pET21a and pET26b, respectively, with signal peptide at the N-terminal for periplasmic expression in *E. coli* (Fig. 1b). The Her2-Fab and Her2-S-Fab were purified by two-step affinity purification, first with Ni–NTA-agarose and then anti-CH1 affinity purification (Fig. 1b). As the VHH is relatively small and soluble, the addition of anti-CD16 VHH did not affect the expression level and solubility of anti-Her2 Fab. The solubility and expression level of Her2-S-Fab were comparable to the control Her2-Fab at 0.6 mg/L.

To determine whether Her2-S-Fab folds correctly as heterodimer, gel filtration was used to analyze the purified proteins. The majority of protein ran as a single peak. The light and heavy chains assembled into intact Fab antibodies with molecular weights of ~65 kD (Fig. 1c) and ~50 kD (data not shown), similar to the expected molecular weights of Her2-S-Fab and Her2-Fab, respectively, suggesting that majority of Her2-S-Fab is correctly folded.

### Her2-S-Fab recognizes HER2 positive cells

**Fig. 2:**
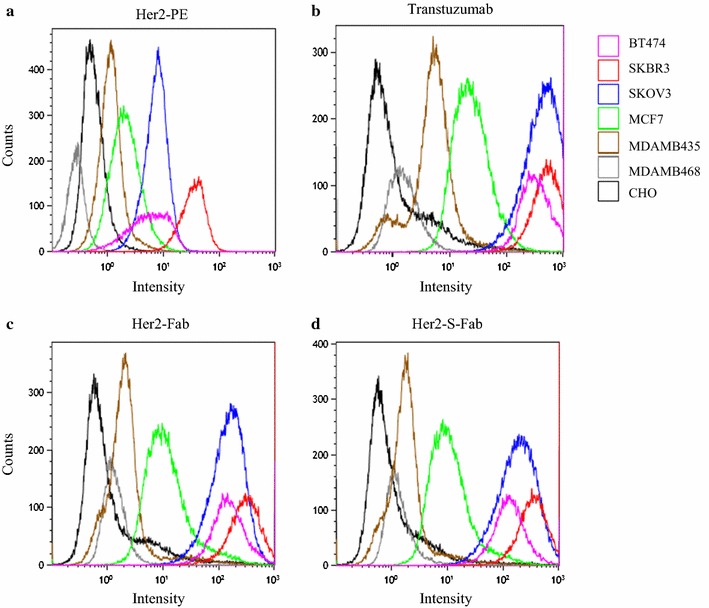
Her2-S-Fab recognizes Her2 positive cells. Flow cytometry analysis of Her2-PE antibody (**a**), Transtuzumab (**b**), Control Fab (**c**), Her2-S-Fab (**d**) on different cancer cells were performed as described in the “Materials and methods” section, *Black line*, CHO cell; *Gray line*, MDAMB435; *Dark blue line*, MDAMB468; *Purple line*, MCF7; *Orange line*, SKBR3; *Light blue line*, SKOV3; *Pink line*, BT474

To check whether Her2-S-Fab can bind to of cells with Her2 expression, flow cytometry analysis was performed using both HER2 positive and HER2-negative cells. In line with previous reports (Lewis et al. 1993; Junttila et al. 2014), using control anti-Her2 antibody, Her2 negative cells CHO, MDAMB435, and MDAMB 468, have very low or no staining; MCF7 has low Her2 expression; while BT474, SKBR3 and SKOV3 cells have high Her2 expression (Fig. 2a).

Similar to the previous work (Han et al. 2015), Transtuzumab can stain HER2 expression cells (Fig. 2b). Both Her2-Fab and Her2-S-Fab, also could bind to HER2 positive cells (Fig. 2c, d) and showed similar fluorescence intensity shifts, suggesting that Her2-S-Fab and control Fab have a similar binding potency. Both Her2-Fab and Her2-S-Fab also showed the same staining pattern to Transtuzumab with less intensity, consistent with the monovalent property of Her2-Fab and Her2-S-Fab.

### Her2-S-Fab induces NK cell-mediated cytotoxicity

**Fig. 3:**
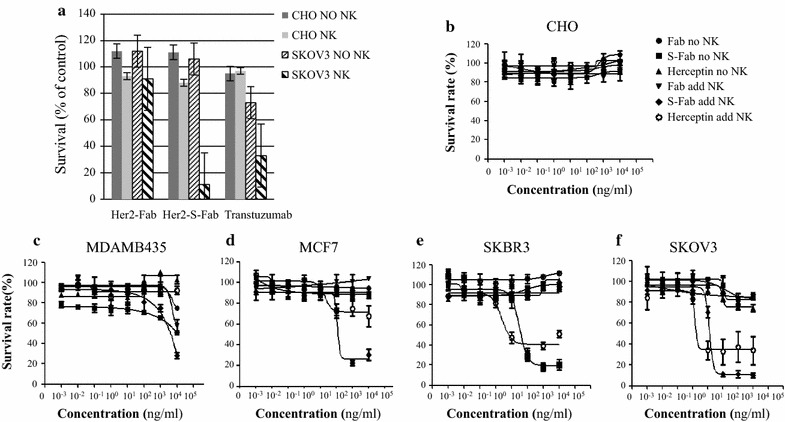
Her2-S-Fab induces NK cell-mediated cytotoxicity. (**a**) Cytotoxicity assay test was performed as described in the “Materials and methods” section for cell lines CHO and SKOV3 with 10 μg/ml of Her2-Fab, Her2-S-Fab or Transtuzumab. The data are the mean of triplicates with *error bars* representing the standard deviation. *Dark*, CHO without NK cells; *grey*, CHO added NK; *dense stripes*, SKOV3 without NK cells; *thin stripes*, SKOV3 added NK cells. **b**–**f** dose dependent cytotoxicity assays were performed as described in the “Materials and methods” section for different cell lines, CHO (**b**), MDAMB435 (**c**), MCF7 (**d**), SKBR3 (**e**), and SKOV3 (**f**). The concentration of Her2-Fab, Her2-S-Fab or Transtuzumab is from 0.01 ng/ml to 10ug/ml. The data are the mean of triplicates with *error bars* representing the standard deviation. *Solid circle*, Fab without NK; *solid square,* Her2-S-Fab without NK; *solid upright triangle*, Transtuzumab without NK; *solid inverted triangle*, Fab added NK; *solid diamond*, Her2-S-Fab added NK; *open circle*, Transtuzumab added NK. NK cells (25,000 per well) and effector cells (2500 per well). The mixtures were incubated for 72 h before cytotoxicity measurement

To evaluate the cytotoxicity of Her-S-Fab, Her2-Fab, Her2-S-Fab, and Transtuzumab were incubated with cancer cells and fresh isolated NK cells. At concentration of 10 μg/ml, no cytotoxicity in the HER2-negative cell line CHO was observed regardless of the presence of NK cell (Fig. 3a). For the HER2-overexpressed cell lines SKOV3, when NK cells are not present, only Transtuzumab decreases the cell proliferation with 72 % of survival rate (Fig. 3a). Her-S-Fab or Her2-Fab has no effect on cell survival. However, in the presence of NK cells, Her2-S-Fab induced potent cytotoxicity, and consistently higher cytotoxicity than Transtuzumab, (survival rate of 10.70 vs. 33.12 %) (Fig. 3a). The cytotoxicity of Her2-S-Fab is dependent on anti-CD16 as Her2-Fab alone has no cytotoxicity even in the presence of NK cells (Fig. 3a).

To further evaluate the activity of Her2-S-Fab on tumor cells, dose-responses of different antibodies on cancer cells were measured. Her2-Fab has no or minimal effect on cell survival regardless of the Her2 expression status (Fig. 3b–f). Transtuzumab only exhibit slight tumor inhibition on Her2 high expression cell SKOV3 without NK cells (Fig. 3b–f). With the presence of NK cells, both Transtuzumab and Her2-S-Fab triggered strong cytotoxicity against SKBR3 and SKOV3 cells in a dose-dependent manner, but had no effect on HER2-negative MDAMB435 and CHO cells (Fig. 3b–f). High concentrations of antibodies were needed to induce cytotoxicity for MCF7, the Her2 low-expression cancer cells, suggesting that the activity of Her2-S-Fab depends on the expression of Her2. In all MCF7, SKBR3 and SKOV3 cells, Her2-S-Fab exhibits stronger cytotoxicity than Transtuzumab, suggesting that Her2-S-Fab is more potent than Transtuzumab.

### Her2-S-Fab inhibits tumor growth in vivo

**Fig. 4:**
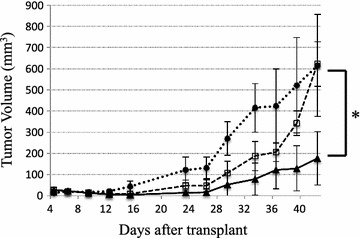
Her2-S-Fab inhibits tumor growth in vivo. NOD/SCID mice (n = 5/group, male) were engrafted subcutaneously with the mixture of SKOV3 cells (2 × 10^6^) and fresh isolated human PBMCs (1 × 10^7^). Two hours later, the mice were then treated with PBS, control Her2-Fab (1 mg/kg) or Her2-S-Fab (1 mg/kg) intraperitoneally for the first dose (D_0_). In the following 6 days (D_1_, D_2_, D_3_, D_4_, D_5_ and D_6_), the mice were administered intraperitoneally (*i.p.*). PBS (*dot line*); Her2-Fab (1 mg/kg, *dash line*); or Her2-S-Fab (1 mg/kg, *solid line*). The data represent the average tumor volume of 5 mice. *Error bars* represent the standard deviation (*P < 0.05, t test, Her2-S-Fab *vs* the other two groups)

An adoptive transfer model was used to test whether Her2-S-Fab can inhibit tumor growth in vivo. SKOV3 cells were first mixed with human PBMCs, and then engrafted subcutaneously into NOD/SCID mice. The mice were then treated with either PBS, Her2-Fab, or Her2-S-Fab. No tumor growth inhibition was observed for tumors treated with Her2-Fab. However, for Her2-S-Fab, strong tumor growth inhibition was observed (Fig. 4).

## Discussion

Cancer immunotherapy has generated great interest due to its potent therapeutic effects on tumors. Among different approaches of cancer immunotherapy, bispecific antibodies have been intensively investigated. In this study, we constructed a bispecific antibody Her2-S-Fab targeting Her2 positive tumor cells. The Her2-S-Fab antibody is linked by the Fab portion of Her2 antibody Transtuzumab and single domain antibody against CD16. The Her2-S-Fab can be produced in large quantity from *E. coli* and exhibit potent cancer cell killing ability comparable or better than Transtuzumab. The cytotoxicity of Her2-S-Fab is specific for HER2-overexpressing cancer cells and depends on the presence of NK cells.

One of the key issues with bispecific antibody is their capacity to trigger an efficient cell-mediated cytotoxicity. Our in vitro data showed a strong and specific NK cell–mediated lysis of Her2-expressing tumor cells. Though only monovalent against Her2, Her2-S-Fab showed stronger cytotoxicity than Transtuzumab with the presence of NK cells (Fig. 3) and PBMCs (data not shown). Different from Transtuzumab, the cytotoxicity of Her2-S-Fab is completely dependent on NK cells (Fig. 3). Though only a few cell lines being tested, similar to other Her2 antibodies, the cytotoxic activity of Her2-S-Fab was affected by Her2 expression levels in cancer cells. The Her2 high-expressing cell lines are more sensitive to Her2-S-Fab than the Her2 low-expressing cell lines. However, more data are needed to generalize the observation.

Single domain antibody was used in the Her-S-Fab due to its physical property. Historically, producing functional and stable bispecific antibodies is difficulty even with various ScFv based formats. These antibodies have the issues of in vitro and in vivo stability and tend to aggregate. In comparison with conventional ScFv, single domain antibody has only half of the size of conventional ScFv, better biophysical properties including high refolding efficiency and high solubility, less tendency for aggregation, resistance to proteases, and high expression in *E. coli*, et al. Thus, it becomes possible to express bispecific antibodies built on single domain antibodies in *E. coli*, as compared with ScFv. As shown in our study, Her2-S-Fab can be produced in and purified from *E. coli* in large quantities (0.6 mg/liter).

Several different formats of anti-Her2 bispecific antibodies have been studied previously, including the IgG formats (Moore et al. 2011; He et al. 2014; Vaishampayan et al. 2015), and tandem ScFv format (Zhou et al. 2015). The IgG formats with Fc have the advantage of prolonged half life in vivo due to the interaction of Fc with FcRn (Covell et al. 1986). However, the production of bispecific antibodies based on such format is still challenging. Smaller size bispecific antibodies based on ScFv or Fab are easier to produce and may have better tumor penetration due to their smaller size. To recruit immune cells, anti-CD3 has been frequently used to recruit T cells in various bispecific antibody formats. However, as activated T cells produce cytokines which may lead to cytokine storm. NK cells have been used as an alternative for targeting tumor cells in the bispecific format. For example, single domain antibody anti-CD16 has been used in a Fab format to recruiting NK cells to target Her2 positive tumor cells (Turini et al. 2014), which two single domain antibodies were used to format a Fab-like antibody. Interestingly, the single domain antibody based Fab-like bispecific antibody also showed increased potency against Her2 positive tumor cells than Transtuzumab (Turini et al. 2014). In our study, we used the same anti-CD16 single domain antibody and combined it with the monovalent of Transtuzumab to target Her2 positive tumor cells.

Comparing to the single domain based Fab-like antibody (Turini et al. 2014), Her2-S-Fab may resemble the human antibody better as natural Fab is used in Her2-s-Fab rather than two camel single domain antibodies used in the Fab-like bispecific antibodies (Turini et al. 2014). As VH-VL interaction can contribute to the assembly of Fab, the production of Her2-S-Fab can be more efficiently. Moreover, as Trastuzumab is specific to Her2 positive cells and has been used extensively in patients, it is potentially safe than other unknown camel anti-Her2 antibodies.

## Conclusion

In summary, the novel bispecific antibody Her2-S-Fab can be used for the redirection of NK cells toward Her2 overexpression cells. Similar to other bispecific antibodies, Her2-S-Fab is efficacious in vitro and in vivo at killing Her2 positive cancer cells. With several formats of bispecific antibodies targeting Her2 positive cancer cells being proposed, it will be interesting to test how the different bispecific antibodies will perform in patient care.
